# Secure Medical Data Model Using Integrated Transformed Paillier and KLEIN Algorithm Encryption Technique with Elephant Herd Optimization for Healthcare Applications

**DOI:** 10.1155/2022/3991295

**Published:** 2022-10-25

**Authors:** Ala Saleh Alluhaidan

**Affiliations:** Department of Information Systems, College of Computer and Information Science, Princess Nourah Bint Abdulrahman University, Riyadh, Saudi Arabia

## Abstract

In the healthcare industry, where concerns are frequently and appropriately focused on saving someone's life, access to interfaces and computer systems storing sensitive data, such as medical records, is crucial to take into account. Medical information has to be secretive and protected by the laws of privacy with restrictions on its access. E-health security is a holistic notion that encompasses available medical data's integrities and confidentiality which ensures that data are not accessed by unauthorized people and allow doctors to offer proper treatment. The patients' data need to be secured on servers holding medical data. This work adds new features for ensuring storage and access safety through ITPKLEIN-EHO (integrated transformed Paillier and KLEIN algorithms) that use EHOs (elephant herd optimizations) to provide lightweight features. The key space affects lightweight encryption techniques in general. The EHOs (elephant herd optimizations) optimize key spaces by adjusting iteration rounds. The main goal is to encrypt EEGs (electroencephalographic signals) in healthcare and send it to end users using the proposed ITPKLEIN-EHO approach. This suggested technique utilizes MATLAB for its tests on various EEG data sets for implementation. The simulations of the proposed IRPKLEIN-EHO technique are evaluated with other existing techniques in terms of MSEs, PSNRs, SSIMs, PRDs, and encryption/decryption times.

## 1. Introduction

**Figure 1:**
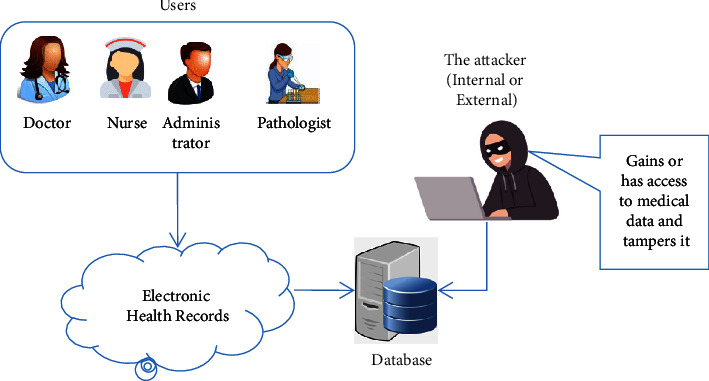
Security risks in traditional electronic health systems.

Daily, more than 7.7 billion people utilize the Internet [1]. Its capabilities have grown to include themes as broad as getting food delivered from one area to another, as well as more vital activities such as money tracking and online banking. The Internet's amicable culture has morphed into one of venom [2] as the number of users has increased. Since the initial data leak, the risks that Internet users have faced have not altered. Inadvertent mistakes (natural and man-made disasters, as well as personnel mishaps) and deliberate operations (fraud, identity theft, embezzlement, and so on) are the two sorts [3]. Numerous breaches of data using the Internet have been the reason for deliberations on the Internet's security aspects that need consideration and resultant secure solutions [4]. Data security has been an important focus of research and discussions in e-health systems since its outset. While data digitalization, increased efficiencies, and speeds have increased the vulnerabilities of data to cyber-attacks, medical records appear to be a popular target for hackers, as seen in Figure 1.

A few information breaks have driven the improvement of hostile to danger security arrangements [5]. Notwithstanding utilizing an assortment of safety methodology including firewalls, VPNs (virtual confidential organizations), and encryptions, mixes of these actions are required for compelling and secure information exchanges. As advancements of sight and sound applications rise, pictures are becoming assumed critical parts in a few applications. E-medical services are one of the most fundamental purposes [6]. Correspondence among specialists and patients has become a lot simpler because of digitization. Specialists from different areas can team up and cooperate. There are various purposes for e-medical care which can anticipate drug collaborations, analyse infections, help medical procedures carefully, and use telemedications/wellbeing. These delicate pieces of information of medical services are sent utilizing public organizations representing an assortment of safety gambles.

Because of the lack of sufficient security measures in place, medical imaging modalities have been targeted by hackers [7]. In today's healthcare information systems, medical data are an essential component of diagnostics [8]. The majority of health institutions maintain their medical data remotely in third-party servers and clouds. For such digital material, privacy, safety, and security must be ensured through the use of encryption to assure secrecy and authentication techniques to ensure authorship. In this arena, digital image data encryption and watermarking solutions had to be reversible [9]. Due to the sensitivity of the data conveyed in medical data, the original plain image data used in cryptography and watermarking methods should be recoverable.

Watermarking is used to introduce identifiers that, by definition, are inseparable from the material in which they are placed. They could be viewed as the ultimate defense against usurpation and falsification. Medical tradition is quite demanding about the quality of biomedical data, and it is frequently forbidden to change the bit field encoding the image in any way (nondestructive) [10]. The data alteration idea underpins the watermarking technology. As a result, the watermarking process must be reversible in the sense that the original pixel values must be precisely retrieved. This severely limits the capability and quantity of approaches available. It also requires dedicated algorithms to suppress and introduce the mark automatically to prevent the delivery of unprotected documents. However, mindlessly applying watermarking is not acceptable in the medical imaging profession, where any change to the image's high information area is not allowed. Encryption is an exceptionally effective method for capacity and transmissions. However, once sensitive data are encrypted, it may not be secure [11] when images are found as open (plain-content) frames where intruders can breach privileges and obtain daily records.

Most typical encryption techniques emphasize text data or paired data [12]. As a result, traditional ciphers such as IDEA, AES, DES, and RSA, among others, cannot encrypt real-time images within specific time durations as they demand longer calculation times and larger registration powers. Considering the advantages of encryptions in protecting data, an effective method of safeguarding medical data was proposed [13]. It has been shown that medical data generally contain confidential information about patients and are thus vulnerable to different security dangers when transmitted over public networks making it compulsory to encrypt images before transmissions over public networks. Traditional encryption approaches, on the other hand, have been found incapable of providing acceptable security levels due to the uniqueness of medical data such as increased correlations and redundancies amongst pixels and bigger sizes. This results in their inability to stand against security threats.

Thus, this work aims to create efficient encryption of images with highly complex secret keys. The heterogeneous network system's nature also takes advantage of (i.e., respirator pumps with a network-permitted system that is distinct from enlisting frameworks that may pay special attention to the Internet). The expanded assessment of sold-off information on under data is set by the growing risk scenario, in which cyber-attacks [14] are becoming increasingly polished and substantially financed. The primary goal is to provide a revolutionary combined transformed Paillier and KLEIN algorithm that makes use of the EHOs. The proposed ITPKLEIN-EHO encrypts EEG signals before their transmissions. The primary contributions of this work are detailed as follows:

- Effective encryptions of images for e-healthcare data using the proposed ITPKLEIN-EHO scheme which secures biological EEG signals with secret keys. The key space affects lightweight encryption techniques in general. The swarm optimization approach is introduced here to optimize key spaces by adjusting iteration rounds.

The remainder of the paper is structured as follows: Section 2 presents related work. The proposed model is discussed in Section 3. The experimental analysis is presented in Section 4.  Section 5 presents the conclusion as well as future work.

## 2. Related Work

The approaches based on encryption and watermarking are addressed in this part to protect medical data for the healthcare system, which provides various significant aspects. Ali et al. [15] suggested a zero-watermarking technique to preserve an individual's privacy and avoid the risk of identifying exposure to telemedicine. The suggested technique embeds a person's identification in medical voice signals without creating any distortion. To select the appropriate sites, two metrics are computed in the signal for identity insertion: Hurst exponent and zero-crossing. Unvoiced speech frames are reliable for identifying insertion and extraction, according to an analysis of the signals as well as resistance to noise attacks. The suggested zero-watermarking approach inserts identifications into secret keys instead of signals using 1-D binary local operators. Kamran and Farooq [16] developed an information-preserving watermarking technique as a restricted optimization problem where the study's experimental results showed diagnostic accuracies while resisting well-known watermark corruption assaults.

Pilot results show that by utilizing the suggested information-preserving strategy, total classification accuracy is never reduced by more than 1%. Vasanthanayaki [17] announced the launch of a new safe and secure system for medical records. The suggested architecture has an impact on cloud infrastructures, allowing for cost efficiency, quick deployment, scalability, and flexibility to meet variable workloads. The research system might be utilized to safeguard a variety of medical documents. The proposed SMCPSs (secure medical healthcare content protection system) have two main steps: HKGs (hybrid key generations) and storage management. Keys and values for healthcare records are initially generated using AES and MD5 (message digest) techniques for secure multiparty computing. The proposed HKG method was distinguished from other key generation algorithms by its use of AES and MD5 in key values. These two methods generate a new key value, increasing the system's security level. The HKGs create dynamic and representative keys for medical records that are computationally efficient to compute and contrast and require little storage. In the second stage of the project, a framework based on compressive sensing (CS) will be developed for the preservation and management of medical healthcare records.

Anand et al. [18] created encrypted dual watermarks for EPR data protection that were compressed using numerous important features. Their experimentations on large medical datasets proved their suggested scheme's viability. Finally, the proposed method outperforms the existing techniques by providing superior robustness and security. The general design of an MCPS by Kocabas et al. [19] consisted of four layers, namely, data collections, aggregations, cloud processes, and actions where the layer's hardware and communication capabilities changed, and separate encryption algorithms were utilized to ensure data privacy in layers. The study examined both traditional and innovative encryption techniques in terms of their capacities to provide secure storage, data shares, and computations. The full experimental evaluation of each strategy reveals that while emerging encryption systems enable exciting new capabilities such as safe sharing and secure computing, they impose computational and storage overhead of several orders of magnitude. The report was concluded by proposing potential research topics for improving the usability of forthcoming encryption techniques in an MCPS. To complete encrypting and decrypting a medical picture, a deep-learning-based picture encryption and decryption networks (DeepEDNs) is used, the method was presented by Ding et al. [20]. Then, cycle-generative adversarial networks (Cycle-GANs) is utilized for shifting medical images from their source domains to target domains and where target domains are “hidden factors” that aided learning models in encrypting data. Encrypted images were restored to their original (plaintext) images through reconstruction networks in decryptions. The scheme eased data mining procedures from privacy-protected environments, ROIs (regions of interest), and mining networks extract relevant items from encrypted images. The chest X-ray test recommended DeepEDNs. Extensive experimental findings and security analysis show that the suggested technique can provide high degrees of security while remaining efficient.

Hasan et al. [21] in their proposal utilized light-weighted encryptions for securing healthcare images. Their suggested lightweight encryption method used two permutations of techniques to protect medical photos. They appraised and compared their approach with generally encrypted schemes in terms of security and execution speed where their performance evaluations on a large number of test photographs showed better outcomes when compared with other existing algorithms in terms of efficiencies.

Yang et al. [22] suggested plaintext encryptions of marked medical images into similar images of target images for increasing image security. Their novel scheme RDH (reversible data hiding) was based on adaptive texture classifications to embed data privacy into medical images to preserve patients' details while improving image qualities. The recommended RDH beats alternative RDH algorithms in the study's extensive tests. Plaintext encryptions reduced attackers' attention spans and improved the security of medical images.

Encryption for medical clouds was suggested by Li et al. [23] where symmetric encryption schemes were dynamically searched for forward and backward privacies, and at the same time, the SEDSSEs' (secure and efficient dynamic searchable symmetric encryptions) approach employed KNNs (k-nearest neighbors) and ABEs (attribute-based encryptions). In the realm of dynamic searchable symmetric encryptions, their two security aspects were crucial though challenging to establish. Key sharing issues that plague kNN-based searchable encryption systems were handled with a better technique. Their techniques were efficient in terms of storage overheads, index constructions, trapdoor creations, and queries, according to their extensive research.

Zhou et al. [24] suggested medical image encryptions based on game theories. Their scheme optimized ROIs with concealed ROI positions. ROIs were pixel-level transformations used in encryptions to allow lossless decryptions and prevent the loss of information in medical pictures. Simultaneously, ROI's position information was appropriately disguised, preventing positional information leakages during transmissions. Furthermore, hyperchaotic QCNNs (quantum cell neural networks) scrambled and diffused ROIs with random sequences. Most crucially, quantitative parameter analyses of ROIs were presented, and game theory achieved the best balance between encryption speeds and encryption security performances.

Yang et al. [25] in their study presented a medical data share method based on attribute crypto-systems and blockchain technologies. In the first place, encoded clinical information was kept up within mists, with capacity locations, and clinical related data were recorded on block-chains, guaranteeing productive capacity and disposing of dangers of irreversible information changes. Second, their recommended framework blended ABEs with ABSs (property-based signs), empowering portions of clinical information in many-to-numerous communications. ABEs guaranteed information security and fine-grained admittance controls, while ABSs confirmed the clinical information's sources while safeguarding underwriter characters. Moreover, information clients reappropriated most clinical information figure text unscrambling exercises to CSPs (cloud specialist co-ops), which significantly diminished handling loads.

Zheng et al. [26] suggested efficient medical data shares that leveraged on ABEs to overcome privacy issues in user data shares. Attribute matching functions were eliminated, and attribute bloom filters were used to conceal the attribute's access control structures. Throughout encryptions, online/offline encryption technologies optimized encryption efficiencies. Before the message was known, a large number of works had to be accomplished at the encryption stages. Cipher texts could be constructed once the message was decoded. Furthermore, at the system's initializations, all characteristics were not needed. The system did not reinitialize when system users' general attributes increased, which enhanced the system's efficiency. Their data shares were secure, according to security and performance studies, and improved data processing capabilities in IoT (Internet of Things)-based data shares.

In a multidata-owner setting, Abdelfattah et al. [27] developed effective searches for encrypted data. The cloud server received noisy similarity ratings, which physicians denoised before downloading most critical articles, to assure the security of the proposed approach. The study's solution allowed doctors to prescribe search conditions without giving them to the server and allowed them to customize searches. Their formal proof and analysis indicated that their system was a safeguard for maintaining privacy against known plaintext and linkability attacks, and their results of several experiments showed that their technique was more efficient than previous methods.

Chen et al. [28] used cloudlet's versatility to construct breakthroughs in the healthcare system. Privacy protections, data interchanges, and intrusion detections are all characteristics of cloudlets. The study adopted NTRUs (number theory research units) to encrypt user-collected physiological data gathered by wearable devices. These data were delivered in an energy-efficient manner to neighboring cloudlets. Secondly, they provided a novel trust model to help users choose trustworthy partners for the exchange of cloudlet data. The trust idea also allowed individuals with comparable diseases to talk to one another about their ailments. Thirdly, the study divided and safeguarded stored medical information into three sections maintained in remote clouds. Their scheme protected healthcare data from malicious assaults using collaborative IDSs (intrusion detection systems) which were based on cloudlet mesh that can prevent clouds from attacks. The tests suggested that the proposed strategy was successful.

Liu et al. [29] described ETC approaches for the analysis of ECG data where data privacy was ensured while reconstructed signals were of the same quality as unencrypted compressions and without sacrificing compression performances. SVDs (singular value decompositions) compressed data specifically for providing quality in encrypted and compressed data. The results of the study's experiments revealed effective ways of data security in addition to enhanced compression performances on ECG data.

POMP, a privacy-preserving medical prediagnosis system for cloud environments created by Yang et al. [22], used LRs (logistic regressions) to provide health care information without invading their privacy. The use of homomorphic encryption methods to provide private prediagnosis procedures on encrypted data was the scheme's distinction. The proposed POMP approach used preprocessing methodologies and Bloom filters to reduce the computing costs of prediagnostics. Through careful research, the study showed that the proposed POMP systems could successfully survive different security concerns and safeguard privacies of information.

Tao and Ling [30] proposed block-chain-based medical file shares based on decentralized ABCs. Authorization applications and grants were recorded on the blockchain. Smart contracts provided system users with interactive platforms. Decentralized ABEs were used to control fine-grained access to medical information, ensuring privacy and security while avoiding single-point failures. The model was brought closer to reality by attribute-based algorithms that permit ted democratic decisions from groups with dynamic personnel changes. Their system worked well in real time in terms of security and practicability while offering new practical models for medical file shares when compared with other similar existing solutions.

Niu et al. [31] proposed permission-based data shares using block-chains. The data's privacies were maintained using cipher text-based attribute encryptions and thus controlled accesses. They employed polynomial equations for building arbitrary connections between keywords and ensuring the safeguard of patient identities where blockchain technology was merged. Furthermore, their random oracle model's keywords were indistinguishable for adaptive chosen keyword assaults. Their system showed high retrieval efficiencies in their analyses.  Table 1 shows a comparison of suggested and current approaches, as well as their advantages and disadvantages.

### 2.1. Inference

According to the study, the majority of the effort is based on the medical image-based security mode; however, it is heavily focused on data security. As a result, this work concentrated solely on medical data. Medical data exchange and storage over public networks are not secure. Authentications, confidentiality, and integrity are the three main challenges with medical data. Medical data security issues can expose patients to several risks, restricting the growth of mobile healthcare apps even more. As a result, medical data must be safeguarded at all times during transmission and storage. Image encryption is one of the information security approaches that may be used to protect medical data.

## 3. Proposed Methodology

**Figure 2:**
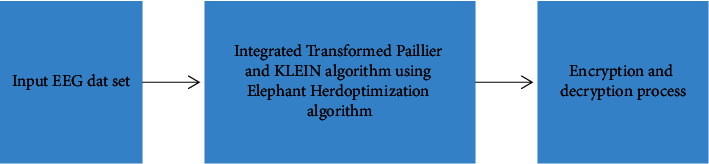
General framework diagram of ITPKLEIN-EHO.

As illustrated in Figure 2, distinct EEG signal data sets are used as a source of data for encryption and decryption. Before being delivered to the hybrid lightweight encryption technique, the data are preprocessed (transformed Paillier and KLEIN algorithm). Transformed Paillier is key pair-based cryptography that adds homomorphism to the system. Because messages are encoded and will interpret as needed, they may be merged in this manner. Every client is given a private and public key, and messages encrypted with the public key may be decoded with the private key. KLEIN is a lightweight encryption estimator that is impacted by key space; it utilizes the EHO improvement framework to minimize key space. This decreases the degree of a cycle and, as a result, the amount of space available. Finally, the EEG signal data have been encoded and unscrambled.

### 3.1. Medical Data Security Model Based on ITPKLEIN-EHO

EEGs are a critical biosignal used by neurosurgeons to handle cerebrum issues. The use of transmission medium and pressure systems to send biomarkers such as ECG and EEG for remote medicinal delivery has improved in the telemedicine industry, boosting realism and testing. To make optimal use of transmission capacity, it is necessary to pack and send this information for the critical treatment/standard healthcare/patient observation framework. In telemedicine applications, transmitting a significant volume of data safely and originally becomes problematic. ITPKLEIN-EHO is presented as a solution to the aforementioned issues.

#### 3.1.1. Transformed Paillier Encryption

Image scaling and cropping in a reduced cloud storage system with deduplication are presented in this section based on two distinct encryption levels. Many users can use this system, but each user (i.e., data owner) wants to utilize two keys to encrypt the image. The key management authority generates these keys for each user by running an initialization process. The initial key pair is used for the first level of encryption that is formed. KLEIN is used by Paillier to encrypt the second key pair. The keys for encryption and decryption are provided by the key management authority. The initialization algorithm represents the initialization of the key management authority. Choose two huge prime integers, pr_1_ and pr_2_, at random and independently, such that gc d(pr_1_*∗*pr_2_(pr_1_ − 1)(pr_2_ − 1))=1. This feature is guaranteed if both primes are of similar length. Computing ‘Sk' for the secret key and gpr_1_ 1 and pr_2_ for the public key, we obtain

(1) $$\begin{array}{c} Sk=pr_{1}*pr_{2}\text{,}\\ \Lambda =lcm\left(pr_{1}-1,pr_{2}-1\right)\text{,}\\ Μ={\left(L\left(g^{\Lambda }\;\mathrm{mod}\;Sk^{2}\right)\right)}^{-1}\;\mathrm{mod}\;Sk\text{.} \end{array}$$

Here, *g* ∈ *Z*, the public keys for encryption are Sk and *g*, and the private keys for decryption are Λ and Μ. Let the image pixel pr_1_ be encrypted, where Λ is the requested leakage function, 0 ≤ pr_1_ ≤ Sk, and a random number rand are chosen from a range of integers, where 0 rand *n*. The encryption procedure will be represented by (2) using these two values. Here, *E* is the encrypted image pixel, min *p* is the minimal polynomial, and rand^*n*^ is an integer random number.

(2) $$\begin{array}{c} E=\min\;p∙rand^{n}\;\mathrm{mod}\;Sk^{2}\text{.} \end{array}$$

After the encryption process is complete, the user will perform the second level of encryption before performing the first level of decryption *D* using the secret key (3), which represents this decryption procedure.

(3) $$\begin{array}{c} D=L\left(c^{\Lambda }\mathrm{mod}\;Sk^{2}\right)∙M\mathrm{mod}\text{,} \end{array}$$

where *D* represents the decoded pixel of the given image and *c* represents the plaintext.

#### 3.1.2. KLEIN Encryption Process

The structure of KLEIN is a conventional substitution-permutation network (SPN), which is used in several sophisticated block cyphers, including AES and PRESENT. To give an acceptable security buffer and asymmetric iteration for KLEIN64/80/96, the rounds counts NR are estimated as of 12/16/20. To extend relatively small master keys into successive round keys and round transformations, all viable block ciphers use distinct key schedules. The key schedule must be flexible, and KLEIN is used to build block cipher-based hash functions and message authentication codes, even when keys are updated often. Key scheduling, on the other hand, considers the right level of security complexity. KLEIN's key scheduling is designed to eliminate potential related-key weaknesses while balancing performance and is detailed as follows:

(1) Input: a 64/80/96 bit master key Mk for KLEIN-64/80/96.
(2) Key scheduling: let *i* be the round counter of KLEIN-64/80/96. In the first round, when *i* = 1, the initial subkey sk_1_ = mk = sk_0_^1^‖sk_1_^1^‖ *·* *·* *·*‖sk_t_^1^, where *t* = 7/9/11 for KLEIN-64/80/96. For KLEIN-64, the (*i* + 1)th subkey sk^*i*+1^ can be derived from the *i*th subkey ski as follows:
  - Divide the *i*th subkey sk_i_ into two byte-oriented tuples, such that *t*_1_ = (sk_0_^*i*^, sk_1_^*i*^, *·* *·* *·*, sk_⌊*t*/2⌋_^*i*^) and *t*_2_ = (sk_⌊*t*/2⌋_^*i*^, sk_⌊*t*/2⌋+1_^*i*^,…, sk_*t*_^*i*^) for the next step
  - Cycling left shift one byte position in (*t*_1_, *t*_2_), obtain t_1_′ = (sk_1_^*i*^, *·* *·* *·* , sk_⌊*t*/2⌋_^*i*^ , sk_0_^*i*^) and *t*_2_′ = (sk_⌊*t*/2⌋+1_^*i*^, *·* *·* *·* , sk_*t*_^*i*^, sk_⌊*t*/2⌋_^*i*^) for the next step
  - Swap the tuple (*t*_1_, *t*_2_) with a Feistel-like structure, such that *t*_1_^″^ = *t*_2_′ becomes the left tuple, whilst *t*_2_^″^ = *t*_1_′ ⊕ *t*_2_′ becomes the right tuple
  - XOR round counter *i* with the third byte in the left tuple *t*_1_^″^, and we substitute the second and the third bytes of the right tuple *t*_2_^″^ by using the KLEIN S-box S
(3) Output: iteratively execute the above step for different key lengths, truncate the leftmost 64 bits of subkey sk_*i*_ for the *i*th round transformation.

**Figure 3:**
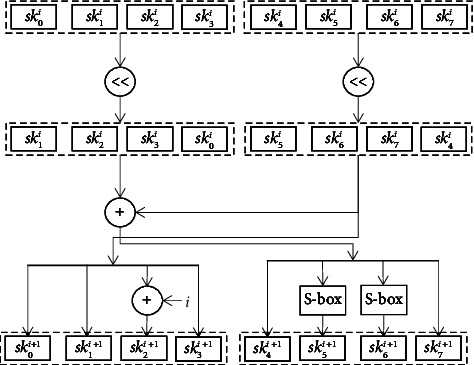
The key schedule algorithm of 64 bit key length.

**Figure 4:**
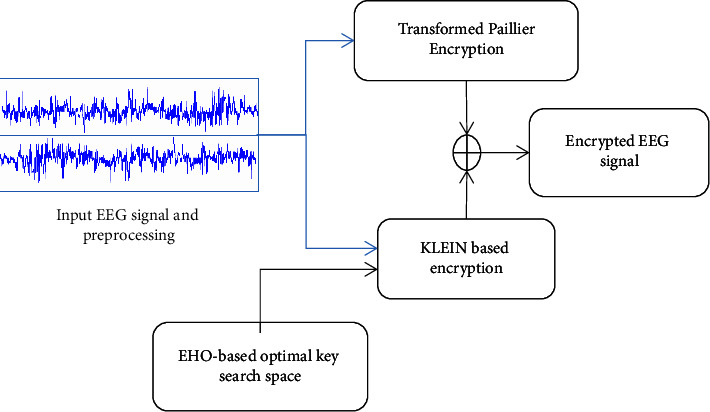
The system model for encryption of the EEG signal.

**Figure 5:**
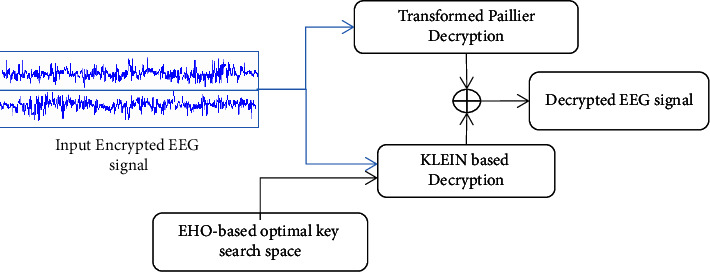
The system model for encryption of the EEG signal.

The KLEIN-64 key schedule technique is shown in Figure 3. Different key sizes can be accommodated using KLEIN's key scheduling. KLEIN subkeys can be generated in round transformations to save memory for retaining interim values. KLEIN's on-the-fly key scheduling is more resource-efficient than classical optimizations, which involve computations of all subkeys beforehand during the sensor's performance tuning. Figures 4 and 5 show the system model for EEG signal encryption and decoding.

#### 3.1.3. Elephant Herding Optimization

By varying the number of iteration rounds, EHO was able to optimize the key space. The authors in [33] proposed the EHO technique, which is an SI technique. It is based on natural elephant herding behavior. This behavior is summarised as follows: elephants are split into clans, each of which has numerous elephants. Each clan is led by a matriarch, while adult male elephants leave their clans and live alone. Two operators in EHO handle these actions: clan update and clan separation. Clan updates move elephants and matriarchs about within each clan, whereas separation enhances population diversity later in the search phase.

**Figure 6:**
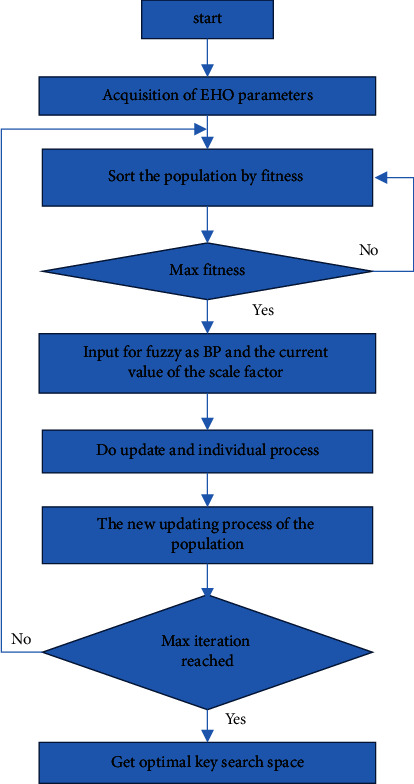
Flowchart of EHO for optimal key space detection.

EHO behavior classifications include separating operators and clan updating operators. As shown in Figure 6, EHO outperforms conventional approaches for detecting critical search spaces. The elephant A matriarch, usually the eldest cow, leads the herd. Each member ‘*j*' of the clan I move following the matriarch, who is the elephant with the highest fitness value in a generation:

(4) $$\begin{array}{c} Fitness=E{lnew}_{cl_{i,j}}=El_{cl_{i,j}}+\alpha \left(El_{best,cl_{i}}\right)\times r\text{.} \end{array}$$

In clan *i*, Elnew_,cl_*i*,*j*__ depicts an elephant *j* in a present location, and El_cl_*i*,*j*__ is its previous location. The best solution for clan cl_*i*_ is the Elbest_cl_*i*__, where *α* ∈ [0,1] is a scale factor of the procedure that recognizes the matriarch position of the best elephant in clan El_bert,cl_*i*__:

(5) $$\begin{array}{c} E{lbest}_{cl_{i}}=\beta \times E{lcenter}_{cl_{i}}\text{.} \end{array}$$

Here, *β* ∈ [0, 1] is the second parameter of the procedure that controls the influence of the Elcenter_cl_*i*__ defined as (clan-updating process):

(6) $$\begin{array}{c} E{lcenter}_{cl_{i}}=\frac{1}{n_{cl_{i}}}\times \overset{{n_{cl}}_{i}}{\underset{j=1}{\sum }}El_{cl_{i},j,d}\text{.} \end{array}$$

Here, 1 ≤ *d* ≤ *D* is the dimension of the *d* th term and denotes the dimension total space, and *n*_cl_*i*__ recognizes an unlimited amount of elephants in a clan *i*. In each clan *i* elephants, which are shifted to the fresh positions as per the following equation (separating process):

(7) $$\begin{array}{c} E{lworst}_{cl_{i}}=El_{\min}+\left(El_{\max}-El_{\min}+1\right)\times rand\text{.} \end{array}$$

Here, El_min_ and El_max_ are the minimum and maximum bounds of the search space, and Rand [0, 1] is a stochastic distribution between 0 and 1.

Each potential solution is tied to a matriarch, which distinguishes EHO from other population-based evolutionary methods. “member” and “clan” are two possible solutions found in the search space. To create the EHO process, an elephant population is formed. Each elephant's matriarch position is then revised based on the elephant's and the clan's experiences. The elephants should seek better alternatives. The objective function of an optimization problem can be used to evaluate the fitness of each elephant, and the input is

(8) $$\begin{array}{c} BP_{El_{i}}=\frac{Fitness\left(E{lbest}_{i}^{k}\right)-Fitness_{KN}}{Fitness\left(E{lbest}_{i}^{1}\right)-Fitness_{KN}}\text{.} \end{array}$$

**Figure 7:**
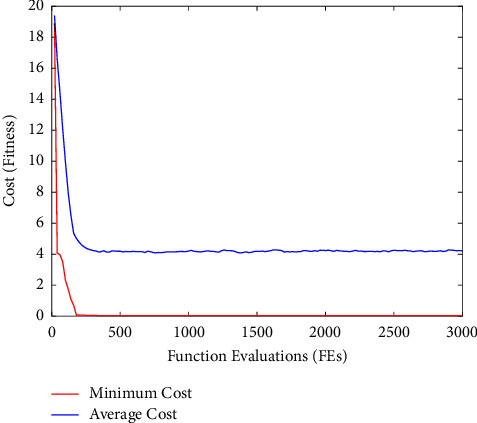
The cost function graphical portrayal of EHO for key search space.

**Figure 8:**
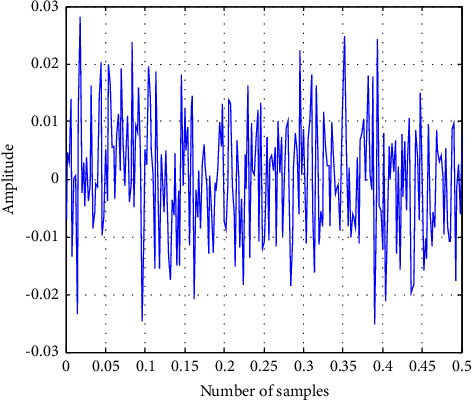
The input of the original EEG signal.

**Figure 9:**
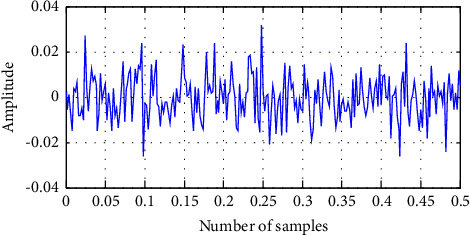
The output of the encrypted EEG signal using ITPKLEIN-EHO.

Fitness(Elbest_*i*_^*k*^) is the fitness of the elephant's best previous position in the kth iteration, Fitness KN is the known genuine optimal solution value, and Fitness(Elbest_*i*_^1^) is the fitness of the elephant in the first iteration. The current value of the scale factor for *i*th elephant El_*i*_ is the second fuzzy input. Here, the longest time of Fitness KN, which is consistent *K* and is the current generation number, is KN, and the biggest generation number is KN.  Figure 7 depicts a cost function graphical representation of EHO for a key search space with an elephant in a unique position and a subsequent new position. Figures 8 and 9 demonstrate the input EEG signal and the encrypted signal output.

## 4. Experimental Results and Discussion

This section details the results of quality measurements and cryptography time for clinical datasets used in a sign, including SSIMs (structural similarity indices), NAEs (normalized absolute errors), QILV (quality index-based local variance), PSNRs (peak signal-to-noise ratios), and MSEs (mean-squared errors). The suggested approach will be implemented in the MATLAB tool, and the results will be compared to current methodologies such as ABE [25] and DeepEDN [20] in terms of a variety of common evaluation metrics to reveal system efficiency. The results of the recommended and current approaches are compared in this section. The plain input EEG signal that was encrypted and the resultant output encrypted EEG signal are shown in Figure 7. The experiment employed a total of 20 ECG signal samples from the MIT-BIH databases. The database has a recognition rate of 100%. This study examines three dissimilarity representations: two first-order spaces, the Euclidean and cosine spaces, and one second-order space, Dinc, which will be addressed later. Due to the inability of typical triangulation techniques to capture the original signal, the inclusion of a second-order dissimilarity metric offers fascinating security benefits. As long as the encoded sign's histograms are distinct, the recommended approach is effective against histogram-based attacks. When the encryption technique is performed, the input signal is referred to as the plain EEG signal or original signal, and it is transformed into an EEG encrypted signal, i.e., output. MSEs determine the differences between unique (*x*) and encoded (*y*) signals of length *N*, as illustrated in the following section.

### 4.1. PSNR

This is commonly used to assess the quality of the encrypted signals. The parameter is defined as the peak signal power-to-noise ratio in the decrypted medical data *S*_dec_. A higher PSNR value implies that the scheme has superior denoising capacity.

(9) $$\begin{array}{c} SNR=\log_{10}\frac{S_{\max}^{2}}{MSE}\text{.} \end{array}$$

### 4.2. NAEs

These parameters show estimated error values from intensity differences where lower values approximating to zero indicate fewer errors in decrypted signals.

(10) $$\begin{array}{c} NAE=\frac{\overset{N}{\underset{i=0}{\sum }}\left|S_{enc}\left(x,y\right)-S_{dec}\left(x,y\right)\right|}{\overset{N}{\underset{i=1}{\sum }}\left|S_{enc}\left(x,y\right)\right|}\text{.} \end{array}$$

### 4.3. MSE

This is a commonly used distortion measure. The parameter estimates the average of the square of errors. The parameter is nonnegative, and values closer to zero are better.

(11) $$\begin{array}{c} MSE=\frac{1}{N}\overset{N}{\underset{i=0}{\sum }}{\left[S_{enc}\left(x_{i},y_{i}\right)-S_{den}\left(x_{i},y_{i}\right)\right]}^{2}\text{.} \end{array}$$

### 4.4. SSIM

The parameter is computed for finding the similarity between the encrypted image *S*_enc_ and the decrypted signal. Its value should be within [0, 1]. A higher value indicates a better-decrypted signal.

(12) $$\begin{array}{c} SSIM=\frac{\left(2\mu_{S_{enc}}\mu_{S_{de\;c}}+v_{1}\right)\left(2\sigma_{S_{enc}}\mu_{S_{de\;n}}+v_{2}\right)}{\left(\mu_{S_{enc}}^{2}+\mu_{S_{de\;n}}^{2}+v_{1}\right)\left(\mu_{S_{enc}}^{2}+\mu_{S_{de\;n}}^{2}+v_{2}\right)}\text{.} \end{array}$$

Here, *μ* is an average of the image, and *σ* are the variances of the images. *v*_1_ and *v*_2_ are two variables for stabilizing the weak denominator.

### 4.5. QILV

This gives a comparison of the local variance distribution of the restored image concerning the pain signal. A higher index value indicates better signal quality.

(13) $$\begin{array}{c} QILV=\frac{2\mu_{S_{enc}}\mu_{S_{de\;c}}}{\mu_{S_{enc}}^{2}+\mu_{S_{de\;c}}^{2}}∙\frac{2\sigma_{S_{enc}}\sigma_{S_{de\;c}}}{\sigma_{S_{enc}}^{2}+\sigma_{S_{de\;c}}^{2}}∙\frac{\sigma_{S_{enc}*c}}{\sigma_{I_{enc}}\sigma_{S_{de\;c}}}\text{.} \end{array}$$

Table 2 shows a comparison of several filtering algorithms. The performance of the ITPKLEIN-EHO-based denoising strategy is shown to be effective among them. The best values of MSE, SSIM, NAE, and PSNR in the table demonstrate this. This could be related to KLEIN's usage of an EHO-based key search space, resulting in nonlocal pixel similarity when leveraging redundant information in the signal. Furthermore, the assessment indices SSIM and QILV provide the best results with this method. This demonstrates improved image detail preservation when decrypting. Furthermore, the ITPKLEIN-EHO-based approaches improve the assessment indices for all parameters. This demonstrates the approach's advantage in maintaining crucial search space in medical data. The best NAE scores suggest higher picture detail registration with fewer inaccuracies.

**Figure 10:**
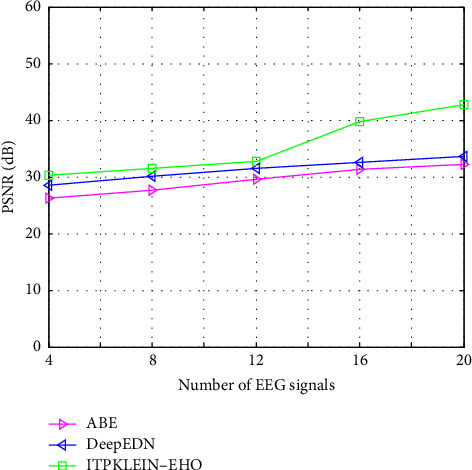
PSNR of different methods compared with the proposed framework.

Figure 10 depicts a comparative examination of several strategies using the proposed model with PSNR. When compared to other techniques, the PSNR value produced by the suggested framework is higher. The proposed approach has the greatest PSNR value (42.68 dB) among the other methods tested, including ABE and DeepEDN. Figure 10 compares the PSNR value for various types of signals using various approaches. The PSNR acquired for various signal levels ranges from 30.25 dB to 42.68 dB. These results show that the ITPKLEIN-EHO is best suited for security models and boosting medical data authentication.

**Figure 11:**
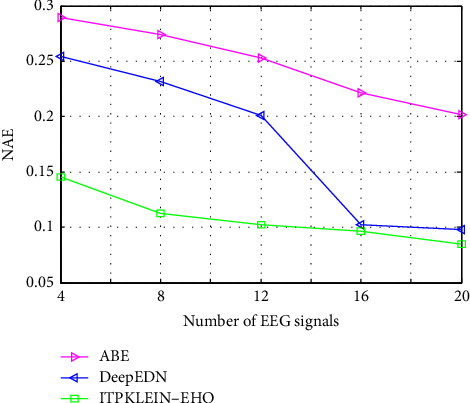
NAE of different methods compared with the proposed framework.

Figure 11 depicts a comparative examination of several strategies using the suggested model with NAE. When compared to other techniques, the suggested framework yields a higher NAE value. The proposed technique has the lowest NAE value (0.0845) among the other methods (ABE and DeepEDN). Furthermore, when compared to other approaches, the NAE values for all types of photos are low. This could be due to KLEIN's EHO-based key search space selection. Furthermore, because of the employment of the security model, it is rotationally invariant.

**Figure 12:**
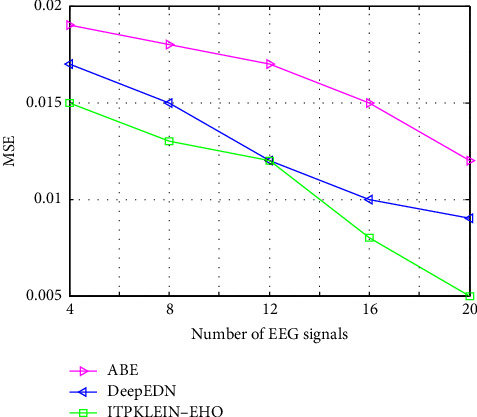
MSE of different methods compared with the proposed framework.

Figure 12 depicts a comparative examination of several strategies using the proposed model with MSE. When compared to other techniques, the MSE value produced by the suggested framework is higher. The proposed approach has an MSE value of 0.005, which is low when compared to other methods such as ABE and DeepEDN. MI values imply improved registration of image features. According to the findings, ITPKLEIN-EHO-based approaches are effective in producing security in medical data. Because the performances of previous approaches are limited due to their computational complexity, the findings of existing methods achieved high NAE.

**Figure 13:**
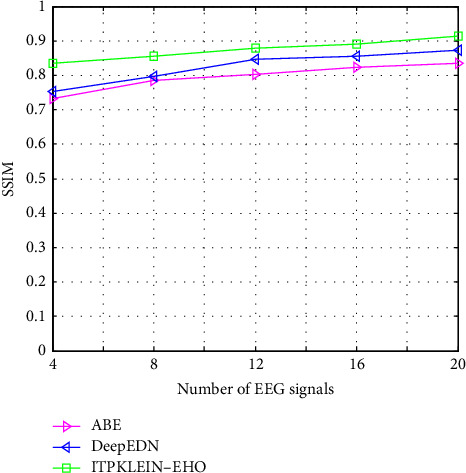
SSIM of different methods compared with the proposed framework.

Figure 13 depicts a comparative examination of several strategies using the suggested model with SSIM. When compared to other techniques, the SSIM value obtained by the suggested framework is higher. The proposed approach has the highest SSIM value of 0.9145 when compared to other methods such as ABE and DeepEDN. The SSIM value obtained for various photos ranges from 0.8358 to 0.9145. The EHO model was created to locate the best key search space based on the optimum location of the elephant with the least amount of effort. This also solves the EHO training techniques' local minima problem.

**Figure 14:**
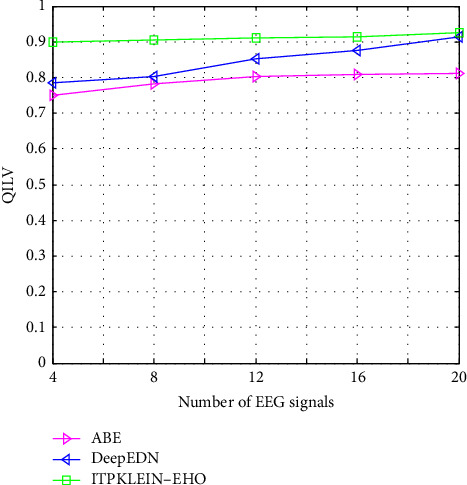
QILV of different methods compared with the proposed framework.

Figure 14 depicts a comparative examination of several strategies using the suggested model with QILV. When compared to other techniques, the suggested framework yields a higher QILV value. The proposed method's QILV value is 0.9254, which is the greatest among the other methods such as ABE and DeepEDN since it finds the optimal KLEIN encryption results with the lowest computational cost. The best values QILV represent the ITPKLEIN-EHO for security production.

### 4.6. Noise Attack Evaluation

Table 3 shows the rate of RMS differentiation (PRD) in cryptograms with disorder developments. The recouped sign loses data when the disturbance is upgraded to the cryptogram by a noise ambush. Furthermore, the enhanced modification effectively counteracts natural outcry when the cryptogram is sent via the dubious channel. Regardless, the suggested encryption approach is vulnerable to a clatter attack discovered by software engineers, and a couple of frameworks to avoid this problem should be addressed.

**Figure 15:**
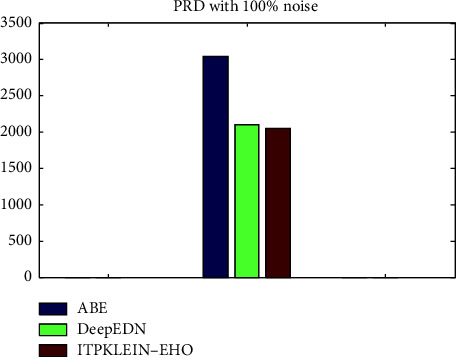
PRD value for the proposed v/s existing method.

In Table 3 and Figure 15, the value obtained in the proposed systems PRD with 100 percent noise was 2056.39. As a result, the suggested encryption scheme generates a competent sign. The encryption method incorporates the modification of the primary clinical sign before encryption. The rate of root-mean-square differentiation (PRD) is used in this criterion to select a certain curvature among prominent clinical signs, and the PRD of the recovered clinical sign is expressed as follows: the message between the patient and the doctor via telemedicine should be progressive. For example, the interaction between the two people groups should be as quick as possible. The encryption and unscrambling procedure in the secure message system requires processing time.

**Figure 16:**
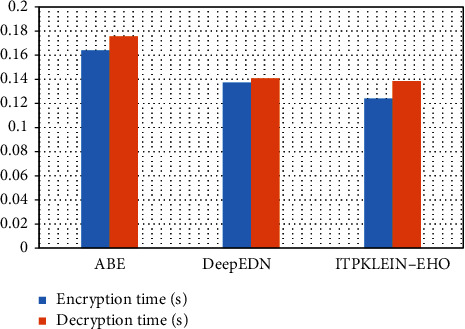
Encryption/decryption times of proposed and existing methods.

The encryption and unscrambling times of the proposed ITPKLEIN-EHO scheme are compared to known techniques for clinical EEG signal datasets in Table 4 and Figure 16. According to the aforementioned Table 4, the proposed method's cryptography timings are 0.1241 s and 0.1385 s. As a result, as compared to the present approach, the suggested ITPKLEIN-EHO method takes less time to process. Finally, the proposed ITPKLEIN-EHO approach outperforms the others.

## 5. Conclusion and Future Work

The proposed ITPKLEIN-EHO method outperformed the existing one in terms of performance. Comparing this strategy to older ones, it produced better outcomes in terms of error reduction and processing time for cryptography as measured by using a simulation tool. According to the experimental results, the proposed approach has a 93.5% accuracy rate, compared to a 91% rate for existing methodologies. As a result, the EEG signal data for each patient can be transmitted safely, quickly, and without incident. In the future, we will work to cut down on the encryption time so that real-time applications can use it. The proposed technique also reduces the expense of keeping electronic health records. The method not only offers fine-grained access control but also prevents adversarial doctors from uploading false data during the authentication procedure. Blockchain technology may also be used in the future to allow medical institutions to exchange digitised health information. Clinical professionals may develop quick and precise diagnosis plans for patients with the help of medical information used effectively, which also boosts hospital productivity.

## Figures and Tables

**Table 1:** A comparative table of proposed and existing methods with their advantages and disadvantages.

Author	Method	Advantages	Disadvantages
Ali et al., [15]	Zero-watermarking algorithm	Increase the encryption and decryption process's security level	Because some areas in this approach are more important in the diagnostic process, slight changes in them might modify the diagnosis

Kamran and Farooq [16]	Information-preserving watermarking scheme	Excellent performance and on-time delivery	The investigation discovered, however, that the present recommended approaches still had application-specific security issues

Vasanthanayaki [17]	SMCPS	The suggested approach has a fast computational speed	The photos of the patients, on the other hand, are not encrypted

Anand et al., [18]	Compression-then-encryption-based dual watermarking	The suggested technique has demonstrated good encryption and decryption performance, as well as minimal complexity	The investigation discovered, however, that the present recommended approaches still had application-specific security issues

Ding et al., [20]	DeepEDN	The suggested approach is simple to implement	The memory, CPU, and energy budgets are all limited

Hasan et al., [21]	Lightweight encryption algorithm	The suggested system's encrypted entropy and encryption speed are high, protecting patients' privacy and boosting the security of medical photos on the cloud	The use of security analytics has the potential to drastically reduce vulnerability concerns.

Yang et al., [22]	RDH	In terms of storage, search, and update difficulty, the approaches are superior	Searching encrypted medical data without decryption, on the other hand, is challenging

Li et al., [23]	SEDSSE	It delivers optimal and lossless picture encryption and decryption, as well as the ability to safeguard medical photos flexibly and reliably	At the low embedding rate, the image contrast does not appear to be boosted

Zhou et al., [24]	A lossless medical image encryption scheme	Reduce the computational load significantly	However, being vulnerable to data manipulation and forgery is a disadvantage

Yang et al., [25], zheng et al., [26], tao and ling [30]	ABE	It protects against known-plaintext/background and linkability attacks while maintaining privacy	However, sharing paper-based medical data between two or more medical institutions was cumbersome and time-consuming

Abdelfattah et al., [27]	Search scheme over encrypted data	Increase the encryption and decryption process's security level	Smart terminals, on the other hand, are typically restricted in computer capacity, and users' privacy concerns persist

Chen et al., [28]	NTRU	Excellent performance and on-time delivery	Users' sensitive information is involved, and communication energy is used

Liu et al., [29]	Singular value decomposition	It necessitated little overheads	This technique, however, has a substantial computing cost since it compares individual keywords in the trapdoor to all of the keywords

Guo et al., [32]	Homomorphic encryption	It features a low computation/communication overhead and a quick search time	However, numerous problems remain in the creation of an online medical prediagnosis system, including medical information leakage and misuse

Niu et al., [31]	Searchable attribute-based encryption	Low computation and transmission overheads are required	However, this results in erroneous search results and the downloading of unnecessary material, which may lead to doctor misdiagnosis

**Table 2:** The numerical results of existing and proposed methods based on parameters such as PSNR (dB), NAE, MSE, SSIM, and QILV.

Number of EEG signals	PSNR (dB)			NAE			MSE			SSIM			QILV		
	ABE	DeepEDN	ITPKLEIN-EHO	ABE	DeepEDN	ITPKLEIN-EHO	ABE	DeepEDN	ITPKLEIN-EHO	ABE	DeepEDN	ITPKLEIN-EHO	ABE	DeepEDN	ITPKLEIN-EHO
4	26.21	28.56	30.25	0.2895	0.2541	0.1456	0.019	0.017	0.015	0.7321	0.7541	0.8358	0.7514	0.7856	0.8992
8	27.56	30.14	31.48	0.2741	0.2314	0.1125	0.018	0.015	0.013	0.7841	0.7956	0.8567	0.7823	0.8015	0.9042
12	29.63	31.56	32.69	0.2531	0.2012	0.1026	0.017	0.012	0.012	0.8014	0.8452	0.8789	0.8024	0.8512	0.9098
16	31.25	32.59	39.65	0.2214	0.1023	0.0962	0.015	0.01	0.008	0.8241	0.8564	0.8896	0.8098	0.8752	0.9124
20	32.15	33.54	42.68	0.2018	0.098	0.0845	0.012	0.009	0.005	0.8362	0.8741	0.9145	0.8125	0.9125	0.9254

**Table 3:** PRD value for the proposed v/s existing method.

Methods	PRD with 100% noise
ABE	3041.6
DeepEDN	2104.4
ITPKLEIN-EHO	2056.39

**Table 4:** Encryption/decryption times of proposed and existing methods.

Methods	Encryption time (s)	Decryption time (s)
ABE	0.1640	0.1756
DeepEDN	0.1372	0.1408
ITPKLEIN-EHO	0.1241	0.1385

## Data Availability

The data are available at https://physionet.org/content/mitdb/1.0.0/.
